# Implications of mitochondrial phosphatidylethanolamine in neuronal health and neurodegeneration

**DOI:** 10.4103/NRR.NRR-D-25-00201

**Published:** 2025-07-05

**Authors:** Yantao Zuo, Niharika Amireddy, Qian Cai

**Affiliations:** Department of Cell Biology and Neuroscience, Division of Life Sciences, School of Arts and Sciences, Rutgers, The State University of New Jersey, Piscataway, NJ, USA

**Keywords:** Alzheimer’s disease, autophagy, cognitive dysfunction, *de novo* phospholipid biosynthesis, hereditary spastic paraplegia, neuronal phospholipid trafficking, Parkinson’s disease, phosphatidylserine decarboxylase, phosphatidylserine transport, tauopathy

## Abstract

Phosphatidylethanolamine is a major phospholipid class abundant in the brain, particularly in the inner leaflet of the plasma and mitochondrial membranes. Although it is primarily synthesized from phosphatidylserine via decarboxylation in mitochondria or from ethanolamine via the cytidine diphosphate-ethanolamine pathway in the endoplasmic reticulum, phosphatidylethanolamine that resides in mitochondria is preferentially produced locally and is distinct and separate from the pool of phosphatidylethanolamine made in the endoplasmic reticulum. Mitochondria-derived phosphatidylethanolamine is not only essential for mitochondrial integrity but also is exported to other organelles to fulfill diverse cellular functions. Neurons are highly enriched with phosphatidylethanolamine, and the importance of phosphatidylethanolamine metabolism in neuronal health has recently been recognized following its reported links to Alzheimer’s disease, Parkinson’s disease, and hereditary spastic paraplegia, among other neurological disorders. Indeed, disturbances in mitochondrial function and phosphatidylethanolamine metabolism and the resulting neuronal dysfunction are the common features of individuals suffering from these diseases, highlighting the great importance of maintaining proper phosphatidylethanolamine homeostasis in neurons. In this review, we summarize the current knowledge of phosphatidylethanolamine metabolism and its role in neuronal function with a special emphasis on the phosphatidylethanolamine biosynthetic pathway in mitochondria. We then review findings on how phosphatidylethanolamine biosynthesis is affected in major neurodegenerative diseases. Finally, we highlight promising future research areas that will help advance the understanding of neuronal phosphatidylethanolamine mechanisms and identify phosphatidylethanolamine-targeted therapeutic strategies for combating such brain diseases.

## Introduction

Phosphatidylethanolamine (PE) is a primary lipid species of eukaryotic cell membranes, second only to phosphatidylcholine (PC) in prevalence. It constitutes approximately 15%–25% of the overall phospholipid composition in cell membranes and is particularly enriched in the brain, accounting for approximately 45% of total phospholipid content relative to approximately 25% in other cell types (Gibellini and Smith, 2010; Vance, 2015; Choi et al., 2018). Exceedingly high quantities of PE are found in the plasma membrane and mitochondria, serving a critical role in maintaining their structural fluidity and integrity (van der Veen et al., 2017). The composition of PE involves a glycerol backbone with two fatty acid chains bonded through ester linkages alongside a phosphate group bonded to an ethanolamine molecule as the hydrophilic head. PE is usually in a lamellar phase in membranes. However, its conical shape and affinity for a non-bilayer structure enable it to adopt non-lamellar phases, particularly the hexagonal II phase, which facilitates cellular fusion and fission processes, protein integration into membranes, as well as induction of conformational changes in protein structure (Dowhan and Bogdanov, 2009; Lessen et al., 2022). Additionally, recent research has uncovered diverse roles of PE beyond membrane fluidity and integrity, encompassing cellular signaling, lipid metabolism, and ferroptosis (Acoba et al., 2020; St Germain et al., 2023).

Neurons are highly polarized cells, and the axons of neurons can extend up to a meter or more from the cell body. Results from some of the first studies support the notion that both the cell body and the axon of neurons have the capacity to synthesize all main membrane phospholipids, including PC, PE, and phosphatidylserine (PS) (Strosznajder et al., 1979; Gould et al., 1983; Vance et al., 1994, 2000). Notably, synaptic vesicle (SV) membranes contain relatively high levels of PE (20%) (Deutsch and Kelly, 1981; Takamori et al., 2006). PE plays a crucial role in neurons, without which the proper functioning of neural membranes and neuronal communication would be compromised. Dysregulation of lipid pathways has been implicated in a growing number of neurodegenerative disorders. Over the past two decades, PE has become a significant research focus and has been increasingly linked to the pathogenesis of neurodegenerative diseases, including Alzheimer’s disease (AD), Parkinson’s disease (PD), and hereditary spastic paraplegia (HSP), which are characterized by progressive degeneration of selective neuron types accompanying functional impairments (Wang et al., 2014; Calzada et al., 2016; Blusztajn and Slack, 2023; Phan et al., 2023). Mitochondrial defects are also a hallmark of these diseases (Cai and Jeong, 2020). Although the extramitochondrial pathways for PE biosynthesis exist, the majority of cellular PE is produced in mitochondria and the endoplasmic reticulum (ER) (Acoba et al., 2020). Importantly, when compared to the whole cell, mitochondria are more abundant in PE, which is mostly derived from imported PS and is pivotal for maintaining mitochondrial integrity and function. In this review, we summarize the existing knowledge on PE metabolism with a focus on the mitochondrial PE biosynthetic pathway along with the main cellular functions involving PE in the neuronal system. We also review PE defects in neurodegenerative disorders and highlight the potential of developing PE-targeted therapeutic strategies warranting future research.

## Search Strategy

We carried out the literature search using the PubMed database from inception to April 2025. Our search was limited to articles published in English. Key words were “Alzheimer’s disease, autophagy, cognitive dysfunction, *de novo* phospholipid biosynthesis, hereditary spastic paraplegia, neuronal phospholipid trafficking, Parkinson’s disease, phosphatidylserine decarboxylase, phosphatidylserine transport, tauopathy.” While we have focused on studies published in the last 15 years, we have also included earlier publications with critically important discoveries.

## Phosphatidylethanolamine Biosynthetic Pathways and Metabolism

**Figure 1:**
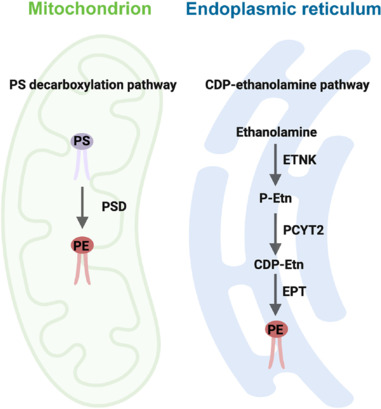
Primary biosynthetic pathways of PE in mammalian cells. The primary routes of PE synthesis include the PSD pathway in mitochondria and the CDP-ethanolamine (Kennedy) pathway in the ER. In mitochondria, PS is decarboxylated to PE by PSD, an enzyme restricted to the inner mitochondrial membrane. PE is also synthesized in the ER through ETNK-mediated phosphorylation of ethanolamine to P-Etn which is converted to CDP-Etn by PCYT2. PE is finally produced via combining CDP-ethanolamine with diacylglycerol by the ER integral membrane protein EPT. Created with BioRender.com. CDP-Etn: CDP-ethanolamine; EPT: CDP-ethanolamine:1,2-diacylglycerol ethanolaminephosphotransferase; ER: endoplasmic reticulum; ETNK: ethanolamine kinase; PCYT2: CTP:phosphoethanolamine cytidylyltransferase; PE: phosphatidylethanolamine; P-Etn: phosphoethanolamine; PS: phosphatidylserine; PSD: phosphatidylserine decarboxylase.

In eukaryotic cells, four distinct metabolic pathways are involved in the formation of PE. However, the major PE production pathways are the phosphatidylserine decarboxylase (PSD) pathway in mitochondria (Borkenhagen et al., 1961; Zborowski et al., 1983; Böttinger et al., 2012; Horvath et al., 2012) and the cytidine diphosphate (CDP)-ethanolamine pathway in the ER (Kennedy and Weiss, 1956; van Hellemond et al., 1994; Mancini et al., 1999; **Figure 1**). Occurring exclusively at the inner mitochondrial membranes (IMM) (Borkenhagen et al., 1961; Percy et al., 1983; Zborowski et al., 1983), mitochondrial PE biosynthesis via the PSD pathway involves decarboxylation of PS. PS, the precursor of PE, is made in the ER or the mitochondria-associated endoplasmic reticulum membrane (MAM), a close contact between the ER and mitochondria that facilitates lipid synthesis and exchange (Hubscher et al., 1959; Kuge et al., 1997; Stone and Vance, 2000; Bergo et al., 2002; Arikketh et al., 2008). Newly made PS then translocates to mitochondria, where it is converted to PE through the catalytic activity of PSD, an IMM-anchored complex composed of α/β subunits produced after autocatalysis (Horvath et al., 2012; Tamura et al., 2012). The *PISD* gene is the only known gene responsible for producing the PSD enzyme in mammals (Zborowski et al., 1983). Importantly, mice with deletion of *PISD* are embryonically lethal, highlighting that the mitochondrial PSD pathway cannot be adequately compensated by extramitochondrial PE pools (Steenbergen et al., 2005). PE made in the PSD pathway is crucial for membrane integrity and the function of mitochondria. As discussed later, some mitochondria-derived PE could be exported into the ER and other membrane organelles, but the underlying mechanisms remain largely unknown.

The CDP-ethanolamine pathway, also known as the Kennedy pathway, mediates PE biosynthesis in the ER using ethanolamine as the substrate that undergoes phosphorylation to form phosphoethanolamine through the action of ethanolamine kinase (Lykidis et al., 2001). Specifically, the catalytic action of CTP:phosphoethanolamine cytidylyltransferase (PCYT2), encoded by the *Pcyt2* gene, yields CDP-ethanolamine (Nakashima et al., 1997). The integral ER membrane protein CDP-ethanolamine:1,2-diacylglycerol ethanolaminephosphotransferase (EPT) then transforms CDP-ethanolamine and diacylglycerol into PE, constituting the final step of PE metabolism in the CDP-ethanolamine pathway. Radioactive labeling studies in isolated rat hepatocytes indicated that the cytidylytransferase reaction in the synthesis of CDP-ethanolamine catalyzed by PCYT2 is a rate-limiting step of this pathway (Sundler and Akesson, 1975b).

Besides the two major PE biosynthetic pathways, small amounts of PE are known to be synthesized through a base-exchange reaction catalyzed by phosphatidylserine synthase 2 (PSS2) in the ER (Stone and Vance, 2000). Additionally, lyso-phosphatidylethanolamine (lyso-PE) can also be converted to PE via acylation by the acyltransferase Ale1 in the ER (Stein and Stein, 1966). However, these two pathways contribute insignificantly to the overall PE production in mammalian cells (Vance, 2015).

### Interplay between the phosphatidylserine decarboxylase and the CDP-ethanolamine pathways

Whereas most of the cellular PE content in mammalian cells is attributed to PE synthesized by both the PSD and CDP-ethanolamine pathways (Calzada et al., 2016), the relative contribution from each of these two major PE biosynthetic pathways varies by cell type. For instance, over 80% of PE is produced through the PSD pathway in BHK-21 cells and Chinese hamster ovary (CHO) cells, even when ethanolamine is available in the culture medium for the CDP-ethanolamine pathway (Voelker, 1984; Miller and Kent, 1986). In contrast, the CDP-ethanolamine pathway was reported to be the major source of PE in rat hepatocytes and the hamster heart (Zelinski and Choy, 1982; Tijburg et al., 1989; Bleijerveld et al., 2007). Thus, cell or tissue type and the specific alterations of catalytic activity of enzymes in the two major PE pathways are among the determinators of the activity of these pathways and their relative importance. In neurons, while membrane PE content is known to originate from both of these two main pathways (Vance et al., 2000), the relative contribution from each of them remains unclear.

PE species were reported to translocate between mitochondria and the ER (Kainu et al., 2013), making it possible for the two pathways to compensate for each other in PE production. While results from early studies in mice have revealed the importance of PE made in both mitochondria and the ER, a complete disruption of PSD or PCYT2 activity in mice cannot be adequately substituted with PE production from the other biosynthetic pathway (Steenbergen et al., 2005; Fullerton et al., 2007, 2009). Furthermore, studies in CHO cells with acute blockage of PSD activity by RNAi and in PSB-2 cells, the mutant CHO cells with chronic decrease in mitochondria-derived PE levels due to a defect in PS synthesis (Saito et al., 1998), have revealed abnormal morphology and functional deficits of mitochondria, accompanied by drastic impairment in cell growth (Tasseva et al., 2013). Reciprocally, mice with deletion of the *Pcyt2* gene displayed embryonic lethality, indicating that mitochondrial supply of PE to the ER failed to fully replace the role of the CDP-ethanolamine pathway (Fullerton et al., 2007, 2009). These findings imply that the PE pools made in mitochondria via the PSD pathway and in the ER via the CDP-ethanolamine pathway are largely compartmentalized and functionally different. This notion was further strengthened by the evidence from some *in vitro* studies. In CHO cells, the majority of mitochondrial PE was found to be produced by the PSD pathway, while essentially no PE in the mitochondrial membranes was imported from the ER (Shiao et al., 1995), indicating that the PE found in mitochondrial membranes is primarily supplied locally from the decarboxylation of PS rather than from the ER CDP-ethanolamine pathway. Another work further assessed the difference in PE species made in mitochondria and the ER in McArdle and CHO-k1 cells through radioactive labeling of pathway-selective isotope precursors followed by high-performance liquid chromatograph-mass spectrometry analysis (Bleijerveld et al., 2007). In line with previous observations, it was noted that while the major PE species synthesized via the CDP-ethanolamine pathway exhibited mono- or di-unsaturated fatty acids on the sn-2 position, e.g., (16:0–18:2) PE and (18:1–18:2) PE, PE species with mainly polyunsaturated fatty acids (PUFAs) on the sn-2 position, e.g., (18:0–20:4) PE and (18:0–20:5) PE in McArdle cells and (18:0–20:4) PE and (18:0–22:6) PE in CHO-K1 cells, were preferably produced from the PSD pathway and preferentially retained in the mitochondrial membranes. However, it is unclear whether PE saturation is altered with the disruption of any of these two pathways.

Interestingly, a recent study has revealed that distinct from mammals, *Drosophila* pect (equivalent to PCYT2 in mammals) mutants lacking the CDP-ethanolamine pathway caused aberrant phospholipid composition and retinal degeneration, while increasing PE synthesis through the PSD pathway was able to fully restore levels and composition of cellular PE, thereby rescuing the mutant phenotypes in a manner dependent on lipid exchange between mitochondria and the ER (Zhao and Wang, 2020). Therefore, the existing evidence supports the notion that the two major PE synthesis pathways provide largely distinct PE pools likely important for phospholipid homeostasis of mitochondria and the ER that serve different roles in cell function and organism development. The extent by which these two organelles can compensate for each other in PE production may vary significantly by organism, cell type, and specific deficits in the PE biosynthesis that is involved. Continued research is clearly warranted for a better understanding of the relationship in PE supply between these organelles under normal and pathological conditions.

### Biosynthesis of the phosphatidylethanolamine precursor phosphatidylserine

**Figure 2:**
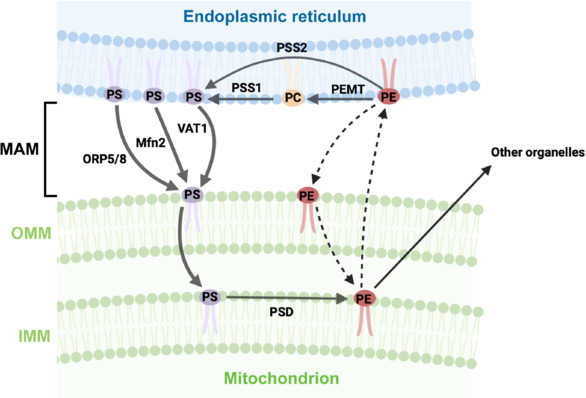
Transport of PS and PE between the ER and mitochondria in mammalian cells. PS is synthesized from PC by two PS synthases, PSS1 and PSS2, in the ER and the MAM, a domain of the ER that is closely juxtaposed to mitochondria. Newly made PS is transported to mitochondria by LTPs, such as ORP5/8, Mfn2, and VAT1 before being imported into the IMM for decarboxylation to PE by PSD. PE derived from imported PS in mitochondria is exported to other organelles, whereas PE exchange between the ER and mitochondria is inefficient. PE is converted to PC by the ER/MAM enzyme PEMT in hepatocytes. Created with BioRender.com. ER: Endoplasmic reticulum; IMM: inner mitochondrial membrane; LTP: lipid transport protein; MAM: mitochondria-associated endoplasmic reticulum membranes; Mfn2: mitofusin 2; OMM: outer mitochondrial membrane; ORP5/8: oxysterol-binding protein-related protein 5 and 8; PC: phosphatidylcholine; PE: phosphatidylethanolamine; PEMT: phosphatidylethanolamine methyltransferase; PS: phosphatidylserine; PSD: phosphatidylserine decarboxylase; PSS1: phosphatidylserine synthase 1; PSS2: phosphatidylserine synthase 2; VAT1: vesicle amine transport 1.

PS is the substrate of PSD and serves as a vital phospholipid constituent of cell membranes, playing critical roles in cell signaling, apoptosis, and membrane structure (Segawa et al., 2014; Zhou et al., 2015; Lenoir et al., 2021). PS is made in the ER membrane or MAM, and newly synthesized PS needs to be transferred from the ER to mitochondria for PS decarboxylation by PSD in the IMM (**Figure 2**). Supported by the findings from radiolabelling studies, it is widely acknowledged that PS translocation from the ER to mitochondria is the rate-limiting step of the PSD pathway (Voelker, 1989; Shiao et al., 1995). PS synthesis in the ER is mediated by phosphatidylserine synthase 1 (PSS1) or PSS2, encoded by PTDSS1 or PTDSS2, through the action of a base-exchange reaction where L-serine replaces either choline in PC or ethanolamine in PE, respectively (Stone and Vance, 2000; Leventis and Grinstein, 2010). It is worth noting that PS synthases are abundant in MAM and a significant number of PS is made in MAM (Stone and Vance, 2000; Kannan et al., 2017).

Specifically, PSS1 primarily utilizes PC as the substrate for PS synthesis and is found in all tissues, with high levels in the brain and skeletal muscle (Suzuki and Kanfer, 1985; Sturbois-Balcerzak et al., 2001). PSS2 mediates PE conversion to PS and is mainly expressed in the testes and contributes minimally to PS production in other tissues (Bergo et al., 2002; Kuge et al., 2003). Earlier studies demonstrated that overexpression of PSS1, but not PSS2, in rat hepatoma cells led to an elevation in PS synthase activity, indicating that PSS1 activity is rate-limiting (Stone and Vance, 1999). Moreover, the knock-down of PTDSS1 appears to inhibit PS decarboxylation to PE (Kainu et al., 2013), whereas enhancement of PSS1-mediated PS synthesis is accompanied by a subsequent increase in PS-derived PE in the IMM (Stone et al., 1998), suggesting that PS biosynthesis and its import to mitochondria are coupled to PE synthesis via the PSD pathway.

Aside from results in cultured cells, both PSS1 and PSS2 appear to play major roles in PS supply in mice, as evidenced by substantial decreases in serine exchange in mice lacking either gene (Bergo et al., 2002; Arikketh et al., 2008). In particular, PSS2 deficiency was shown to lead to a 90%–95% reduction in serine exchange without altering the overall phospholipid content. Such defects were associated with increased choline exchange, suggesting that PSS1 activity might have compensated for decreased PS synthesis by PSS2. However, there was no corresponding increase in PTDSS1 mRNA expression, implying the likelihood that other mechanisms, such as a reduction in PS degradation, also help maintain the phospholipid levels (Bergo et al., 2002). In PTDSS1-deficient mice, total serine exchange activity dropped by 85%. Surprisingly, PS levels remained unchanged in most tissues except for the liver of these mice (Arikketh et al., 2008). Based on these findings, it would be valuable to further determine whether PSD-mediated PS-to-PE conversion is affected in mice with deletion of PTDSS1 or PTDSS2, which could provide important *in vivo* insights into how PE homeostasis is maintained in response to compromised catalytic activity of PSS1 or PSS2. Besides, PS biosynthetic activity has been indicated in neuronal cell bodies and axons (Gould et al., 1983; Vance et al., 1994). However, the underlying mechanisms and the impact on mitochondrial PE biosynthesis remain unknown in neurons.

### Phosphatidylethanolamine in phosphatidylcholine biosynthesis

Although it is inefficient, mitochondria-made PE can be exported to the ER for the synthesis of PC (**Figure 2**), the substrate of PSS1 and a key phospholipid in eukaryotic cells, which constitutes 40%–50% of the total phospholipids in most organelles (Vance, 2015; van der Veen et al., 2017). PE is known to be trimethylated to form PC, which accounts for 20%–30% of PC in liver cells (Sundler and Akesson, 1975a; DeLong et al., 1999). This is a minor pathway for PC production relative to the CDP-choline pathway in the ER, where CDP-choline condenses with a diacylglycerol moiety to produce PC (Kennedy and Weiss, 1956; Bremer et al., 1960; van der Veen et al., 2017). This minor pathway occurs in the ER and MAM and could utilize PE from any of the four PE biosynthesis routes discussed above (Vance, 2015). This reaction is driven by phosphatidylethanolamine N-methyltransferase (PEMT), an enzyme highly expressed in liver cells (Vance, 2014). The conversion of PE to PC is vital for maintaining PC homeostasis in liver membranes, especially when dietary choline intake is low. The PC/PE ratio affects membrane integrity, and proper PC balance has been shown to be crucial for liver health, with imbalances linked to liver diseases (Li et al., 2006). Specifically, patients with chronic liver diseases associated with steatohepatitis displayed a decrease in the PC/PE ratio. A reduction in this ratio was also indicated in mouse and rat models of alcoholic fatty liver disease (Kharbanda et al., 2007; Ji et al., 2008). Reductions in PC content or the PC/PE ratio were also linked to ulcerative colitis, a chronic inflammatory bowel disease characterized by inflammation and ulcers in the lining of the colon (Braun et al., 2009). Additionally, a growing body of evidence indicates that the content of PC and PE or the PC/PE ratio may influence mitochondrial bioenergetic activity, whereas dysregulation of this ratio has been implicated in metabolic disorders, including insulin resistance, atherosclerosis, and obesity (van der Veen et al., 2017). However, existing knowledge concerning the impact of the imbalance of PC and PE content or the PC/PE ratio in neuronal cells remains very limited.

Importantly, PEMT has been detected at low activity in rat brains and the bovine caudate nucleus (Blusztajn et al., 1979; Mozzi and Porcellati, 1979), hinting at multiple mechanisms that exist to meet choline requirements by *de novo* synthesis in the mammalian brain. Further research has shown that, even though PEMT activity is significantly lower in neurons than in liver cells, it is crucial for normal hippocampal development (Vance et al., 1994). Evidence for this includes findings from PEMT knockout mice, which exhibit increased neuronal apoptosis and reduced levels of calretinin, a neuronal differentiation marker, in the hippocampus (da Costa et al., 2010). Collectively, these observations indicate that PE serves as an essential precursor for the biosynthesis of PC and, thus, PS in the brain, thereby supporting its overall function for the health of neurons.

## Inter-organelle Phospholipid Transport for Phosphatidylethanolamine Biosynthesis

Phospholipid trafficking is a tightly regulated process essential for maintaining membrane integrity, signaling pathways, and overall cellular function (van der Veen et al., 2017). To exert their functions, newly synthesized phospholipids in the ER must be transported to various cellular membranous compartments, such as the plasma membrane, mitochondria, and the Golgi apparatus (Chung et al., 2015; Bevers and Williamson, 2016; Valm et al., 2017; Shai et al., 2018; Monteiro-Cardoso et al., 2022). Pertinent to the PE biosynthetic pathway in mitochondria, studies in CHO-k1 cells labeled with [3H] serine or [3H] ethanolamine have revealed that newly synthesized PS in the ER/MAM needs to be imported to mitochondria, which requires adenosine triphosphate (ATP) and is the rate-limiting step for mitochondrial PE biosynthesis (Voelker, 1989; Shiao et al., 1995).

### Transfer of phosphatidylserine from the endoplasmic reticulum to mitochondria for phosphatidylethanolamine biosynthesis

Several lipid transport proteins have been indicated to participate in PS translocation to mitochondria (**Figure 2**). Recent studies supported a mechanism that facilitates PS transport from the ER to mitochondria through the interaction of ORP5/ORP8 proteins, localized at MAM, with the outer mitochondrial membrane protein PTPIP51 and mitochondrial intermembrane space bridging/mitochondrial contact sites and cristae junction organizing system complexes that bridge the two mitochondrial membranes (Galmes et al., 2016; Monteiro-Cardoso et al., 2022). This interaction enables ORP5/ORP8 to mediate the non-vesicular transport of PS lipids to mitochondria by cooperating with the mitochondrial intermembrane space bridging/mitochondrial contact sites and cristae junction organizing system complexes. Consistent with previous observations in PSB-2 cells lacking PSD activity (Tasseva et al., 2013), depletion of ORP5/ORP8 not only impairs mitochondrial PE biosynthesis but also alters mitochondrial morphology and function (Monteiro-Cardoso et al., 2022), suggesting that ORP5/ORP8-mediated PS transport is critical for maintaining PE homeostasis and the integrity of mitochondria. In addition to its essential role in maintaining mitochondrial dynamics, recent work has uncovered that the mitochondrial protein mitofusin 2 (Mfn2) may also facilitate the trafficking of PS from the ER to mitochondria (Hernández-Alvarez et al., 2019). Mfn2 is highly concentrated in MAM and favors PS transfer to mitochondria and mitochondrial PE metabolism by directly and specifically binding to PS. Interestingly, the Mfn2 homolog Mfn1 showed no capacity to bind PS and generate PS-rich domains in membranes. Moreover, Mfn2 deficiency in the liver led to defects in ER-to-mitochondrial PS transfer and triggered liver disease development (Hernández-Alvarez et al., 2019). Using Xenopus egg components, a study reconstituted PS transport from the ER to mitochondria and identified the cytosolic protein VAT-1 that can stimulate PS transport into mitochondria (Junker and Rapoport, 2015). Notably, the depletion of VAT-1 from the cytosol sufficiently reduced the conversion of PS to PE, highlighting that VAT-1 is crucial for PS transport. As these findings were obtained from the study of non-neuronal cells/systems, it remains poorly understood how such mechanisms operate to facilitate ER-made PS import into mitochondria in neurons.

Notably, by depleting cellular ATP or adding exogenous ATP in permeabilized cell lines, earlier studies have shown that PS import from the ER/MAM to mitochondria is an ATP-dependent process (Voelker, 1984, 1989, 1991; Shiao et al., 1995). Our recent work in primary cortical neurons and mouse brains has demonstrated that PS translocation to mitochondria for PSD-mediated PS conversion to PE in the IMM is powered by mitochondrial bioenergetics (Jia et al., 2025). We employed a fluorescent analog of PS—18:1-06:0 N-(7-nitrobenz-2-oxa-1,3-diazol-4-yl) phosphatidylserine (NBD-PS) in live neurons and monitored PE metabolism/biosynthesis through the PSD pathway in mitochondria. After a brief appearance in the ER, NBD-PS is imported into mitochondria, and its conversion to NBD-PE allows the NBD fluorescent signals to be stabilized in mitochondria (Sleight and Pagano, 1985; Hailey et al., 2010). We found that, in neurons with stimulated anaplerotic metabolism by replenishing tricarboxylic acid cycle intermediates, enhanced oxidative phosphorylation (OXPHOS) led to effective increases in PS transport and, thus, PE synthesis in the mitochondria (Jia et al., 2025). These findings highlight the critical role of OXPHOS in the regulation of mitochondrial PE metabolism/biosynthesis in neurons and further suggest that defects in energy metabolism may disrupt PS transport and, thus, PE production in mitochondria. Therefore, disturbance of PE metabolism in mitochondria could be the consequence of mitochondrial dysfunction, a characteristic feature of neurodegenerative diseases.

### Transfer of phosphatidylethanolamine between the endoplasmic reticulum and mitochondria

PE derived from imported PS is exported from mitochondria to other organelles, such as the ER, plasma membrane, and autophagosomes (Vance, 1991; Hailey et al., 2010; Kainu et al., 2013; Jia et al., 2025). The import of PE into the ER from mitochondria is indicated to mostly occur at the MAM where it facilitates general lipid exchange between the ER and mitochondria (Acoba et al., 2020). As a result, mitochondria-derived PE may serve as the substrate for PC or PS biosynthesis in the ER, as discussed above. It is worth noting that PE is a main component of the autophagosome membrane (Rockenfeller et al., 2015), and several studies have unveiled that PE biosynthesis, either in the ER or in mitochondria, can provide PE for autophagosome biogenesis in non-neuronal cells under starvation conditions (Hailey et al., 2010; Rockenfeller et al., 2015). In neurons, we have recently documented that mitochondrial bioenergetics stimulates the PE supply of mitochondria, but not the ER, for the biogenesis of autophagosomes (Jia et al., 2025). Notably, in accord with the fact that PE in the mitochondrial membranes is preferentially produced via the PSD pathway rather than the CDP-ethanolamine pathway in the ER, results from the studies in mammalian cells and yeast have also shown that PE made in the ER is inadequately imported into mitochondria or can only reach mitochondria to a limited extent through unidentified trafficking mechanisms (Yaffe and Kennedy, 1983; Shiao et al., 1995; Kainu et al., 2013; Calzada et al., 2019). Moreover, previous studies reported that ER-made PE transfers to mitochondria far more slowly than PC (Yaffe and Kennedy, 1983), implying the existence of distinct import mechanisms for different lipid classes.

### Neuronal mechanisms of phosphatidylethanolamine biosynthesis and transport

**Figure 3:**
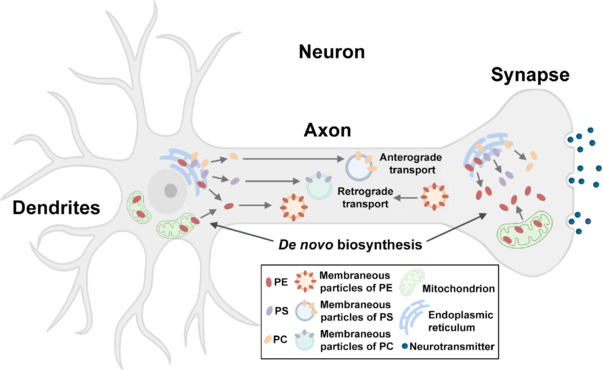
Phospholipid synthesis and transport in neurons. In neurons, all major membrane lipids can be synthesized in the cell body, where PE is produced via the PSD pathway in mitochondria and the CDP-ethanolamine pathway in the ER. Distal axons are also capable of making major phospholipids, including PE synthesis via the two primary routes. Newly synthesized lipids in the cell body are transported in membranous moving particles with various transport kinetics down axons to synaptic terminals by unknown mechanisms. PE produced in axonal terminals also undergoes retrograde transport toward the cell body of neurons. Created with BioRender.com. ER: Endoplasmic reticulum; PC: phosphatidylcholine; PE: phosphatidylethanolamine; PS: phosphatidylserine; PSD: phosphatidylserine decarboxylase.

Neurons are uniquely polarized cells composed of a complex dendritic arbor and a long axon that emerges from the soma and can extend more than a meter in the human body. Therefore, sophisticated mechanisms that allow the efficient biosynthesis and constant supply of newly synthesized proteins and lipids are of great importance for neuronal function and survival (**Figure 3**). In fact, compared to the knowledge established in non-neuronal cells, the current understanding of the mechanism underlying phospholipid biosynthesis in neurons remains very limited (Vance et al., 2000). Early work demonstrated the activities of several enzymes involved in glycosphingolipid biosynthesis and phospholipase D in an axolemma-enriched fraction from the rat brain stem (Costantino-Ceccarini et al., 1979; DeVries et al., 1983). Notably, some lipid biosynthetic enzymes were also identified in myelin, including those for all three steps of the CDP-ethanolamine pathway for PE synthesis and the CDP-choline pathway for PC synthesis in the ER (Kunishita and Ledeen, 1984). Moreover, the activities of EPT, the enzyme responsible for the final step of PE biosynthesis in the ER, were also identified in isolated rat brain synaptosomes (Strosznajder et al., 1979). Thus, while the biosynthesis of membrane phospholipids is highly expected in the cell body of neurons (Vance et al., 1994), these findings highlight the potential of *de novo* lipid production in axonal terminals. Indeed, using the squid giant axon and extruded axoplasm from the giant axon, some further studies provided hints of the biosynthesis of PE from the CDP-ethanolamine pathway and PC from the CDP-choline pathway, respectively (Gould et al., 1983; Tanaka et al., 1987). More evidence, as revealed by employing a compartmentalized microfluidic culture system that allows the separation of the axons from the cell bodies in rat sympathetic neurons, has shown that the distal axon of these neurons is capable of making all major membrane phospholipids, including PE from PS via decarboxylation in mitochondria and by the CDP-ethanolamine pathway in the ER (Vance et al., 1991, 1994). Besides, our recent work in cultured cortical neurons has provided direct imaging evidence showing local PE metabolism/biosynthesis in mitochondria occurs in distal axons, which regulates autophagy activity for autophagic cargo clearance in neurons (Jia et al., 2025). Thus, more investigations are needed to delineate the role of PE and other phospholipid classes in organelle function and neuronal health.

Despite *de novo* PE biosynthesis, phospholipids were also shown to undergo anterograde transport from the cell body toward the axon. Specifically, researchers injected radiolabeled precursors of PE into the L-5 dorsal root ganglion of adult rats and then examined radioactive PE movement in rat sciatic nerves. They found that PE, PC, and cholesterol were transported in rapidly moving membranous particles down axons (Toews et al., 1983, 1988). However, the transport kinetics of these lipids markedly varied, suggesting that different types of vesicles, moving at various speeds, might be responsible for the transport of individual phospholipid classes, including PE (**Figure 3**). This hypothesis was later validated and generally supported by a mathematical modeling analysis (Blum et al., 1992). However, the mechanism underlying the anterograde transport of PE and the interplay of *de novo* PE synthesis in the cell body and axons are currently unknown.

## Phosphatidylethanolamine Functions in the Cell

### Phosphatidylethanolamine in mitochondrial morphology, dynamics, and function

The concentration of PE is higher in the mitochondria relative to the PE content in the entire cell, especially in the IMM, where it accounts for up to 35%–40% of total phospholipids, indicating that PE plays an important role in mitochondrial morphology and function (van Meer et al., 2008; Vance, 2015; Acoba et al., 2020). This is supported by studies in *PISD* knockout mice demonstrating that the elimination of mitochondrial PE production causes fragmented, misshapen mitochondria (Steenbergen et al., 2005). Besides, in PSB-2 mammalian cells, a moderate reduction of mitochondrial PE through suppressing the PSD pathway was shown to result in reversible alterations of mitochondrial morphology (e.g., extensive fragmentation and aberrant formation of respiratory supercomplexes) and function (e.g., decreases in respiratory capacity, ATP production, and cell growth) (Tasseva et al., 2013). These findings highlight the critical role of PE in maintaining mitochondrial structural integrity and function. Mitochondria are dynamic organelles that go through fusion and fission events, the balance of which is important for their health and cellular homeostasis (Campos et al., 2022). The specific mechanisms whereby PE deficiency causes mitochondrial fragmentation or excessive fission remain to be elucidated. Importantly, accumulating evidence indicates that mitochondrial PE levels are decreased with age in humans and animal models (Dai et al., 2021). Such a decline in PE can compromise mitochondrial integrity and may contribute to age-related mitochondrial deficits. Therefore, further research may explore the potential of PE supplementation or interventions targeting PE metabolism for improving mitochondrial health and potentially extending lifespan.

While the detailed mechanisms by which mitochondrial PE influences respiration are yet to be fully understood, existing evidence has implicated several routes. First, by its induction of negative curvature in the IMM (Vance, 2015), PE is instrumental in increasing the surface area of the membrane and thus provides the capacity for more electron-transport chain proteins to bind, leading to increases in mitochondrial respiration (Joubert and Puff, 2021). Second, PE has been shown to affect the function of the respiratory enzyme complexes. Decreased PE in yeast mitochondria is linked to the reduced function of cytochrome c oxidase (complex IV) in the electron transport chain, which is necessary for generating the electrochemical proton gradient in the IMM to drive the ATP-producing oxidative phosphorylation (Böttinger et al., 2012). In addition, *psd1Δ psd2Δ* yeast was shown to have significantly decreased function of cytochrome c oxidoreductase (complex III) and complex IV. While complex IV function was rescued by treatment with ethanolamine, complex III function was not, suggesting that complex III relies on PE synthesized in the IMM (Calzada et al., 2019). Moreover, in mammalian PSB-2 cells, a reduction in mitochondrial PE was found to impair the formation of complex I and complex IV (Tasseva et al., 2013). Another route by which mitochondrial PE might regulate respiration is through its impact on the translocase of the outer membrane complex. Mitochondrial PE depletion impairs the ability of the translocase of the outer membrane complex to import and assemble β-barrel proteins that reside in the outer mitochondrial membrane (Becker et al., 2013). One of these proteins, porin, has been linked to the import of metabolite carrier proteins into the IMM for respiration (Dihanich et al., 1987; Ellenrieder et al., 2019). Taken together, these lines of evidence corroborate the importance of PE in mitochondrial respiration.

### Phosphatidylethanolamine in autophagy

Autophagy is a highly conserved catabolic process within eukaryotic cells, which involves the breakdown of organelles, proteins, macromolecules, and intracellular pathogens to maintain cellular homeostasis (Parzych and Klionsky, 2014; Gatica et al., 2015). A hallmark of autophagy is the formation of double-membrane autophagosomes through, the generation, expansion, and enclosure of a pre-autophagosomal membrane (i.e., phagophore or isolation membrane) (Cai and Ganesan, 2022; Nixon and Rubinsztein, 2024). The supply of phospholipids is a limiting factor for autophagy activity because the life cycle of autophagosomes requires the organized accretion of phospholipids at the nucleation site, and the lipid sources for autophagosome biogenesis have been a focus of recent research endeavors. Growing evidence indicates that PE, a major constituent of the autophagosome membrane, is critically involved in autophagy initiation and autophagosome membrane expansion (Rockenfeller et al., 2015). Compelling data from manipulation of the PE biosynthetic pathways have shown that upregulation of Psd1 markedly increases autophagy and extends lifespan in yeast. Moreover, ethanolamine administration to mammalian cells leads to a rise in PE levels and elevated autophagic flux (Rockenfeller et al., 2015). These observations collectively highlight the critical role of PE in the regulation of autophagy. In addition, PE also functions as an anchor to enable the association of microtubule-associated protein 1A/1B-light chain 3, a member of the autophagy-related protein 8 family, with the autophagosome membrane, which is required for *de novo* autophagosome biogenesis (Ichimura et al., 2000; Tanida et al., 2004; Kabeya et al., 2009). Therefore, reductions in cellular PE compromise autophagosome formation and consequently restrict the availability of autophagosomes for autophagic cargo clearance, which need to be sequestered within autophagosomes to allow autophagy to progress (Rockenfeller et al., 2015; Wilson-Zbinden et al., 2015). While PE biosynthesis either in the ER or in mitochondria was shown to provide PE for autophagosome biogenesis in starved non-neuronal cells (Hailey et al., 2010; Rockenfeller et al., 2015), studies in dorsal root ganglion neurons have reported that the ER was the primary source of membrane supply for autophagosomes (Maday and Holzbaur, 2014). In contrast, results from our recent work in cortical neurons support the notion that OXPHOS-enhanced autophagosome biogenesis and autophagic flux rely on the PE synthesis via the PSD pathway in mitochondria but not the CDP-ethanolamine pathway in the ER (Jia et al., 2025). Thus, apart from the role of the ER in autophagy in dorsal root ganglion neurons, our findings have uncovered, for the first time, that PE made in mitochondria is critically involved in neuronal autophagy regulation.

### Phosphatidylethanolamine in ferroptosis

Ferroptosis is an iron-dependent type of programmed cell death characterized by increased levels of oxidized phospholipids that disrupt cell membranes and ultimately trigger cell death (Tang et al., 2020). As autophagy is involved in ferroptosis, it is also described as an autophagy-related cell death process. Specifically, autophagy can modulate ferritin degradation as well as iron-dependent lipid peroxidation and reactive oxygen species during ferroptosis (Gao et al., 2016; Zhou et al., 2019). However, autophagy is not a primary mechanism of ferroptosis, as it is evident that autophagy inhibitors fail to rescue ferroptosis (Lee et al., 2023; Chen et al., 2024). Given a high content of PUFAs in their membranes, neurons are particularly vulnerable to ferroptosis (Jacquemyn et al., 2024). PUFAs are easily oxidized, and the peroxidation of PUFA-containing phospholipids in neuronal membranes is a characteristic feature of ferroptosis (Ralhan et al., 2023). Iron is also critical for ferroptosis, as it is involved in the process of forming lipid peroxides. As neurons are enriched in iron, iron dysregulation can contribute to ferroptosis, apart from aberrant accumulation of oxidized phospholipids (Snyder and Wu, 2023). While the precise mechanisms and physiological implications of ferroptosis are only partially understood, a growing body of evidence has implicated the contribution of ferroptosis to the pathogenesis of several diseases, including various neurodegenerative diseases (e.g., AD and PD) (Reichert et al., 2020; Yan et al., 2021; Benarroch, 2023). Lipid peroxidation and iron dysregulation, two hallmarks of ferroptosis, have been demonstrated in AD brains (Weiland et al., 2019). Iron dysregulation has also been indicated in PD, and ferroptosis might contribute to neurodegeneration in this disorder (Ryan et al., 2023).

Distinct from classical modes of cell death, ferroptosis can be induced by activation of non-heme iron-containing lipoxygenases and deficiency of a glutathione peroxidase 4 (GPX4), a selenoprotein and antioxidant enzyme that eliminates oxidized phospholipids (Stockwell et al., 2017; Chen et al., 2024). Lipoxygenases are known to mediate lipid peroxidation because free PUFAs are favored lipoxygenase substrates (Li et al., 2022). GPX4 is unique in its ability to reduce lipid hydroperoxides in biological membranes, and its insufficiency is now recognized as a critical factor in ferroptosis (Ursini et al., 1982; Yang and Stockwell, 2016). Without adequate GPX4 activity, cells cannot efficiently mitigate the oxidative damage to lipids, leading to cell death. GPX4 was shown to be inactivated in ferroptosis, triggering lipid peroxidation (Jacquemyn et al., 2024). Additionally, excessive glutamate can deplete glutathione, a critical antioxidant, thereby contributing to ferroptosis (Jacquemyn et al., 2024). Notably, lipofuscin, which contains lipid peroxides and irons, is commonly detected to accumulate in neurons during aging and neurodegenerative diseases (Jacquemyn et al., 2024). Taken together, neurons have multiple mechanisms that can prevent lipid peroxidation, such as GPX4, but these defenses might be compromised in aged and diseased neurons, leading to ferroptosis.

Importantly, redox lipidomic analyses have further shed light on the process, revealing that among all phospholipids, the accumulation of oxygenated arachidonoyl (AA)- or adrenoyl (AdA)-containing PE species, which are key phospholipids during ferroptosis, acts as the immediate trigger for ferroptotic cell death (D’Herde and Krysko, 2017; Kagan et al., 2017). This finding underscores the role of specific lipid peroxidation events in the execution of ferroptosis, with certain PE species being central to the process. Moreover, oxidized PE, specifically 1-stearoyl-2-15-HpETE-sn-glycero-3-PE (SAPE-OOH), serves as a primary phagocytic signal on the surface of ferroptotic cells. This oxidized PE interacts with the Toll-like receptor 2 on macrophages, prompting them to engage in phagocytosis and clear these ferroptotic cells (Luo et al., 2021). Combined, PE and PE plasmalogens are pivotal targets for lipid peroxidation, critical for initiating ferroptosis. Further research on the mechanisms of ferroptosis and molecular details of their involvement in neuronal death in disease is urgently needed because understanding the mechanisms of ferroptosis will open opportunities for the development of novel therapeutic strategies to prevent neurons from ferroptotic cell death.

### Role of phosphatidylethanolamine and phosphatidylserine in neuronal function

Mounting evidence has demonstrated that the synaptic plasma membrane, olfactory bulb, and hippocampus of rat and mouse brains contain markedly higher levels of PE and PS than those in non-neuronal tissues, such as the adrenal glands and the liver (Cotman et al., 1969; Breckenridge et al., 1972; Hamilton et al., 2000; Murthy et al., 2002; Steenbergen et al., 2006), implying that PE and PS may play an important role in neurons. However, the involvement of PE in neuronal aspects has been understudied in the field. The specific contributions of mitochondria *versus* ER-derived PE in neurons and their roles in neuronal maintenance are largely unknown. PE is a main constituent of SV membranes (Westhead, 1987). Given its role in forming and stabilizing membrane structures and promoting membrane fusion (Glaser and Gross, 1994), PE may modulate SV trafficking and recycling and thus facilitate synaptic transmission. Surprisingly, these long-standing questions as to whether and how PE participates in these processes remain unanswered in neurobiology. Previous studies have shown that PE synthesized in both the PSD and CDP-ethanolamine pathways participates in neurite outgrowth induced by nerve growth factor in rat pheochromocytoma (PC12) cells (Ikemoto et al., 1999; Ikemoto and Okuyama, 2000). Relative to PE, PS, the precursor of PE, has been broadly studied in the brain. PS has a high content of docosahexaenoic acid (DHA), an omega-3 fatty acid essential for brain development. PS levels are high in the brain, where DHA is abundant. Conversely, when DHA is depleted from the brain, PS levels decrease (Hamilton et al., 2000). DHA can promote PS synthesis and expand the PS pool in neuronal membranes, thus affecting PS-related signaling that supports neuronal survival and differentiation (Kim et al., 2014). The reduction in DHA levels within PS has been linked to cognitive impairment. Specifically, a decrease in hippocampal PS DHA content was noted in 12-month-old senescence-accelerated prone mice, which exhibit a shorter lifespan, deficits in learning and memory, and increased hippocampal amyloid-β (Aβ) content (Petursdottir et al., 2007). Despite this reduction in DHA corresponding with a rise in arachidonic acid content in hippocampal PS, the potential connection between DHA reduction in PS and cognitive impairment remains to be elucidated. Also, whether depletion of PE containing DHA may affect PS biosynthesis in the brain and thereby lead to memory and cognitive impairment is still an open question. Notably, dietary PS supplements were reported to improve cognitive function in humans and experimental animals (Drago et al., 1981; Corwin et al., 1985; Delwaide et al., 1986). Several changes were demonstrated after the administration of PS, including an increase in neurons positive for brain-derived neurotrophic factor and insulin-like growth factor in the hippocampal CA1 region (Casamenti et al., 1979; Casamenti et al., 1991; Park et al., 2013). However, whether these responses under PS supplementation are attributed to PS-induced effects on neuronal membrane properties and whether such effects are related to PS-derived PE species need to be further investigated. Addressing these questions will provide mechanistic insights into the functional significance of neuronal PS and PE and facilitate validating the therapeutic potentials of PS or PE administration for neurological disorders associated with PS and PE defects and cognitive impairment.

## Phosphatidylethanolamine Abnormalities in Neurodegenerative Diseases

Phospholipid homeostasis in membranes is crucial for cellular signaling and functioning. PE affects membrane protein stability and function and is involved in a variety of cellular processes, such as autophagy, ferroptosis, and inflammation, thereby contributing to pathogenesis (Luo et al., 2009; Segawa et al., 2014). As a matter of fact, mice deficient in either of the two major PE biosynthetic pathways are embryonically lethal (Steenbergen et al., 2005; Fullerton et al., 2007, 2009), suggesting that a disruption in either of these major PE-producing pathways leads to significant defects in PE metabolism and can be pathological. Notably, a decline in PE levels has also been observed during aging in both *Caenorhabditis elegans* (*C. elegans*) and mice (Braun et al., 2016; Gao et al., 2017). However, there is no further evidence concerning alterations and the underlying mechanisms in the PSD or CDP-ethanolamine pathway during normal aging. Given that PE serves as a structural component of neuronal and SV membranes, aged neurons with PE reduction could be more susceptible to insults under pathophysiological conditions, thus exacerbating neuropathology and functional defects associated with age-related neurological disorders such as AD and PD (Calzada et al., 2016). As for neurodegenerative diseases, distinct patterns of PE metabolic dysregulation have been observed, accompanied by different pathologies (Wang et al., 2014; Blusztajn and Slack, 2023). More importantly, emerging evidence suggests that targeting PE metabolism holds therapeutic potential for treating neurodegenerative disorders. Given that mild defects that trigger an alteration in PE abundance are linked to AD, PD, and HSP (**Table 1**), we will highlight the emerging roles of PE in these diseases in the following sessions.

**Table 1:** Summary of PE, PS, and PC findings in AD, PD, and HSP

	Organism/system assessed	Analytic method	PE	PS	PC	Reference
**AD**	Brain tissues of human patients vs. controls	HPLC	Decreased in cortices	No significant change	Decreased in cortices	Nitsch et al., 1992
	Brain tissues of human patients vs. controls	TLC	Decreased in hippocampus and inferior parietal cortex	No data	No significant change	Prasad et al., 1998
	Brain cortices of human patients vs. controls	LC-MS/MS	Decreased	Decreased	No data	Akyol et al., 2021
	Brain tissue of human patients with Lewy bodies vs. controls	LC-MS	No significant change in amygdala	Increased in amygdala	Increased in amygdala	Fu et al., 2022
	Dorsolateral prefrontal cortex of human patients	UHPLC-MS/MS	Decreased	No significant change	Decreased	Batra et al., 2023; Blusztajn and Slack, 2023
	Plasma of human patients vs. controls	LC-MS; NMR	No data	No data	Decreases in PC species (16:0/20.5; 16:0/22.6; 18:0/22.6)	Whiley et al., 2014
	Serum of human patients vs. controls	LC-MS	Decreased	No data	No data	Llano and Devanarayan, 2021
	Brain tissues of PLB4 hBACE1 knock-in mice vs. wide-type	GC-MS	Decreased in the cortex and hippocampus	No change in the cortex and hippocampus	Increased in hippocampus and hypothalamus	Dey et al., 2020
**PD**	Substantia nigra of human patients vs. controls	TLC	Decreased	No significant change	Decreased	Riekkinen et al., 1975
	Visual cortex of human patients vs. controls	LC-MS	Decreased	Increased	No significant change	Cheng et al., 2011
	Frontal cortex lipid rafts of human patients vs. controls	GC-FID	Increased	Increased	No significant change	Fabelo et al., 2011
	Frontal cortex of human patients vs. controls	HRMS	No data	Increased	Decreased	Wood et al., 2018
	Brain tissues of human patients vs. controls	LC-MS	No significant change in amygdala	Increased in amygdala	No significant change in amygdala	Fu et al., 2022
	Plasma of human patients vs. controls	LC-MS	Decreased	No data	Decreased	Chang et al., 2022
	Serum of human patients vs. controls	HPLC-MS	Increased	No significant change	Increased	López de Frutos et al., 2022
**HSP**	Skin fibroblasts of human patients with EPT1 mutation vs. controls	LC-MS/MS	Decreased	No significant change	No significant change	Horibata et al., 2018
	Skin fibroblasts from human patients with PCYT2 variants vs. controls	HPLC-MS	Decreased	Decreased	Decreased	Vaz et al., 2019
	Brain tissues of mice with SELENOI deficiency in nervous system vs. controls	LC-MS/MS	Decreased	No significant change	Increased	Nunes et al., 2024
	Yeast cells expressing SELENOI/EPT1 p.(Pro45Leu) variant vs. controls	TLC	Decreased	No data	Decreased	Kaiyrzhanov et al., 2021

AD: Alzheimer’s disease; EPT1: ethanolamine phosphotransferase 1; GC-FID: gas chromatography with flame ionization detection; GC-MS: gas chromatography-mass spectrometry; hBACE1: human β-site amyloid precursor protein cleavage enzyme 1; HPLC: high-performance liquid chromatography; HPLC-MS: high-performance liquid chromatography-mass spectrometry; HRMS: high-resolution mass spectrometry; HSP: hereditary spastic paraplegia; LC-MS: liquid chromatography-mass spectrometry; LC-MS/MS: liquid chromatography-tandem mass spectrometry; NMR: nuclear magnetic resonance; PC: phosphatidylcholine; PCYT2: CTP:phosphoethanolamine cytidylyltransferase; PD: Parkinson’s disease; PE: phosphatidylethanolamine; PS: phosphatidylserine; SELENOI: selenoprotein I; TLC: thin layer chromatography; UHPLC-MS/MS: ultra-high performance liquid chromatography coupled with tandem mass spectrometry.

### Alzheimer’s disease

With progressive, devastating cognitive decline and neuronal death, AD constitutes the most prevalent neurodegenerative disorder. AD patient brains are characterized by abnormal accumulation of Aβ peptides and hyperphosphorylated tau protein (Long and Holtzman, 2019). While the mechanisms leading to these pathological hallmarks remain to be fully understood, recent studies have implicated dysregulation of lipid metabolism coupled with altered phospholipids, such as PE, PS, and PC, in AD brains (Bennett et al., 2013; Kosicek and Hecimovic, 2013; Akyol et al., 2021). Specifically, notable decreases in the levels of PE and PC were observed in the postmortem brain tissue of AD patients, accompanied by elevated phospholipid deacylation product glycerophosphocholine, as compared to healthy controls. These changes were detected in selective brain regions, including the frontal, primary auditory, and parietal cortices (Nitsch et al., 1992). In line with these observations, a recent comprehensive analysis of over 500 cerebral cortical autopsy specimens from human patients uncovered a robust breakdown of PE and PC as a prominent metabolic deficiency in AD (Batra et al., 2023; Blusztajn and Slack, 2023). Quantitative lipidomics analysis of the brains of the well-known PLB4 AD mouse model with targeted knock-in of human BACE1 (hBACE1) in neurons on an endogenous mouse BACE1 background revealed significant region-specific lipid alterations (Dey et al., 2020). In this model, PE content was reduced within the cortex and hippocampus, consistently suggesting misregulation of PE metabolism in AD. As for PS in the brains of AD patients, some studies reported an increase or no change (Nitsch et al., 1992; Fu et al., 2022), while others found a decrease in PS levels (Akyol et al., 2021). PS levels were also shown to be reduced in PLB4 AD mouse brains (Dey et al., 2020). Additionally, in brain tissues from AD patients compared to individuals with no cognitive impairment, DHA content in PS was lower by 12% in the superior temporal cortex and by 14% in the mid-frontal cortex (Cunnane et al., 2012). Inconsistent results concerning the changes in PS were also obtained in the brains of AD animal models (Ma et al., 2022). Such mixed observations could be attributed to many factors, including distinct brain regions and disease conditions studied (**Table 1**). In AD, PEs were shown to be one of the earliest phospholipids affected with the onset of the disease, and PE levels are known to be reduced by 10% to 30% in gray matter as the disease progresses (Han et al., 2001). Besides, low levels of PE in serum were indicated to be associated with a faster progression from mild cognitive impairment to AD (Llano and Devanarayan, 2021), supporting the possibility that serum PE could be used as a biomarker to predict mild cognitive impairment to AD conversion. As for PC, researchers have identified three PC molecules (PC 16:0/20:5, 16:0/22:6, and 18:0/22:6), which were significantly reduced in the plasma of AD cases compared with those in controls. Furthermore, the overall trend (i.e., control > mild cognitive impairment > AD) suggested a specific link between PC decrease and cognition decline (Whiley et al., 2014).

Aside from the changes in total PE levels, a decrease in PE-PUFAs has been indicated in AD patient brains (Prasad et al., 1998). The following studies have mostly focused on docosahexaenoic acid (DHA, 22:6n-3), a long-chain omega-3 PUFA that is highly enriched in brain tissue, particularly in PE, and is essential for brain development, learning, and vision. Indeed, PE-DHAs are the most abundant, thus constituting the primary storage of DHA in the brain (Sun et al., 2018; Hachem and Nacir, 2022). In the frontal cortex of AD patient brains, DHA content was 28% lower in the lipid raft microdomains, which are specialized regions of the cell membrane enriched with lipids, compared to those in the age-matched control group (Martín et al., 2010). Marked reductions of monoene 18:1n-9 and 20:4n-6 were also detected in these AD lipid rafts, accompanied by increased saturates/n-3 ratio and reduced unsaturation index values that could be attributable to decreases in n-3 long-chain PUFA and n-9 monounsaturated fatty acids. Importantly, such changes augment APP and BACE1 interactions in lipid rafts. Also, decreased contents of n-3 long-chain PUFA likely enhance BACE1 cleavage of APP to promote amyloid burden at the early stages of AD (Fabelo et al., 2014). These ﬁndings are in accord with the results from earlier studies in other brain areas, including the hippocampus of AD patients, where DHA in main DHA-containing PE is drastically reduced (Söderberg et al., 1991; Prasad et al., 1998). Given that phospholipids with PUFAs are susceptible to oxidation (Santos et al., 2024), these observations imply the possibility of oxidative stress-enhanced breakdown of phospholipids in AD. In fact, recent work revealed accelerated turnover of PE and PC as a predominant metabolic defect in AD patient brains (Batra et al., 2023; Blusztajn and Slack, 2023).

As a major phospholipid found in mitochondrial membranes, PE participates in the maintenance of membrane structure (Acoba et al., 2020). Alterations in PE content affect the fluidity and stability of the membrane and the assembly of supercomplexes in the membrane, contributing to changes in mitochondrial morphology and function (Decker and Funai, 2024). In accordance with this, studies from AD-related hippocampal neurons derived from McGill-R-Thy1-APP transgenic rats revealed alterations in mitochondrial lipid profiles, including reductions in PE and PC, which was coupled with impaired mitochondrial bioenergetics (Martino Adami et al., 2019). In a cellular AD model (SH-SY5Y APPswedish transfected cells), mitochondrial lipids containing three and four times unsaturated fatty acids (FA X:4), such as arachidonic-acid, were found to be elevated, while FA X:6 or X:5, such as eicosapentaenoic acid or DHA, showed a decrease (Kurokin et al., 2021). Our recent work in tauopathy mouse brain mitochondria revealed decreases in PE and PS levels, accompanied by defects in mitochondrial bioenergetics (Jia et al., 2025). Reductions in some PE species were also indicated in synaptic mitochondria isolated from 3XTg-AD mouse brains (Monteiro-Cardoso et al., 2015). In contrast to the observations from studies in cellular and animal models of AD, AD patient brains exhibited increases in PC and PE levels in mitochondrial membrane fractions (Jin et al., 2006). As such, further research is needed to advance the understanding of the complex interplay between mitochondrial function, PE metabolism, and the pathophysiology of AD.

Alterations in phospholipids may change the viscosity of cell membranes, thereby hampering enzyme activity, signal transduction efficiency, and the functionality of membrane carriers (Akyol et al., 2021). Aβ peptide is derived from sequential proteolytic cleavages of APP by β- and γ-secretases (Haass and Selkoe, 2007). PE has been indicated to play a role in the regulation of Aβ production by modulating γ-secretase activity. Studies using an *in vitro* system have revealed that PE inhibits γ-secretase activity in a dose-dependent manner (Onodera et al., 2015), whereas other groups using mammalian cells and *Drosophila melanogaster* have shown that when PE levels are reduced, γ-secretase activity is also decreased (Nesic et al., 2012). Hence, less Aβ accumulates in these models with depletion of PE, indicating that PE levels correlate strongly with γ-secretase cleavage of APP for Aβ generation. Given that PE regulates the activities of certain membrane proteins by affecting their topology (Dowhan and Bogdanov, 2009), whether and how the changes in PE content in the membrane influence the accessibility of γ-secretase to APP cleavage site and thus Aβ production needs to be investigated. Additionally, it is unclear how to reconcile such a mechanism and the findings of PE reduction and amyloid accumulation in AD brains.

Presenilin 1 or presenilin 2 functions as the catalytic subunit of γ-secretase, an intramembranous protease, which processes APP to generate Aβ following the prior cleavage by β-secretase (Wolfe et al., 1999; Tanzi and Bertram, 2005). Several studies have implied that familial AD-linked mutations in the PSEN1 gene encoding presenilin 1 or presenilin 2 lead to a tighter association between the ER and mitochondria (Area-Gomez et al., 2009, 2012). Such defects trigger elevations in mitochondrial PE production and the abundance of PE in membranes due to increases in the activity of resident enzymes in both compartments, including PSD (Area-Gomez et al., 2012). The rise in PE levels consequently augments γ-secretase activity and amyloidogenic processing of APP. Besides, a recent study in cultured neural cells and AD mice provided direct evidence demonstrating that Aβ generation is controlled in a MAM gap width-dependent manner, and tight MAM can significantly increase Aβ production (Zellmer et al., 2024). While these findings are consistent with the observations from *Drosophila melanogaster* (Nesic et al., 2012), the mechanism underlying increased ER-mitochondria apposition linked to mutations in presenilin 1 or presenilin 2 remains unknown.

Abnormal accumulation of hyperphosphorylated tau proteins in the cytoplasm, leading to the formation of tau aggregates and fibrils, is a pathogenic hallmark of tauopathy diseases, including AD. Autophagy stress and the presence of tau within autophagosomes are salient characteristics in the brains of AD and other tauopathies (Piras et al., 2016). We recently reported defects in the PE biosynthetic pathway of mitochondria in tauopathy mouse brains, as evidenced by decreases in PE and PS levels (Jia et al., 2025). Strikingly, anaplerotic stimulation of OXPHOS corrected PE metabolism and biosynthesis in mitochondria and enhanced autophagy activity for pathological tau clearance in tauopathy neurons and mouse brains. Such rescue effects were accompanied by attenuations of tau pathology and cognitive impairment in tauopathy mice. Therefore, these findings highlight the pivotal role of mitochondrial bioenergetic dysfunction in PE defects and autophagy failure, which contribute to tau-mediated neurotoxicity and cognitive deficits in tauopathy. Additionally, mounting evidence has indicated that tau is also found in the extracellular milieu, and tau interaction with membranes is essential for both tau secretion and uptake, as well as aggregation (Bok et al., 2021). Hence, dysregulation of PE biosynthesis and the resulting PE imbalance in the cell membrane might influence tau aggregation and propagation by altering tau interaction with membranes, but more research is definitely needed to address this question. Given that we and others have demonstrated mitochondrial energy metabolism deficits as an early feature in tauopathy preceding synaptic dysfunction and tau pathology (Dumont et al., 2011; Kopeikina et al., 2011; Lopez-Gonzalez et al., 2015; Jeong et al., 2022), our study suggests a new therapeutic strategy by early stimulation of OXPHOS to prevent the buildup of pathological tau in AD and other tauopathy diseases. Furthermore, strategies targeting specific enzymes involved in PE metabolism or restoring PE homeostasis can also be evaluated for their effects on AD-related pathology and cognitive deficits to determine their therapeutic potential.

### Parkinson’s disease

Affecting millions of individuals globally, PD is another major progressive neurodegenerative disorder with common symptoms comprising motor problems (e.g., tremors and slowed movements) and non-motor disturbances in cognition, mood, sleep, and other functions (Váradi, 2020). While the progressive degeneration of dopaminergic neurons in the substantia nigra pars compacta and the formation of neuronal α-synuclein (α-syn) aggregates are prominent features of PD patient brains (Simon et al., 2020), the cause of PD remains unclear. Growing evidence has implicated a potential role of altered lipid metabolism in PD-related pathologies. Recent lipidomics studies reported changes in PE, PC, or PS coupled with alterations in other phospholipids in the plasma of individuals with PD, thus supporting the role of such lipids in PD development and progression, possibly as putative biomarkers (Chang et al., 2022; López de Frutos et al., 2022; Qin et al., 2024); however, contradictory results have been reported, which showed increases in PE and PC, but no change in PS in PD patient serum (López de Frutos et al., 2022), or increased PE and PS in the frontal cortex lipid rafts from PD patients (Fabelo et al., 2011). Decreases in total PE levels have been seen in the substantia nigra and the primary visual cortex of PD patient brains or fibroblasts from PD patients (Riekkinen et al., 1975; Cheng et al., 2011; Fu et al., 2022). Notably, PCYT2, the rate-limiting enzyme for PE synthesis via the CDP-ethanolamine pathway, is elevated in the substantia nigra of PD patients (Ross et al., 2001). Thus, convergent evidence indicates that decreased levels of PE in the crucial substantia nigra brain region of PD patients may result in compensatory effects on increased PS levels or a rise in PE biosynthetic enzyme activity in the ER.

PUFA levels were reported to be increased in the brains of PD and dementia with Lewy bodies (Sharon et al., 2003). In this study, researchers further demonstrated that PUFAs can promote α-syn oligomerization and subsequent neuronal damage. Moreover, α-syn was observed to interact with lipid aldehydes, including 4-hydroxy-2-nonenal, one of the main products of DHA peroxidation, consequently enhancing the formation of toxic α-syn oligomers, oligomerization of monomeric α-syn, or stabilization of α-syn oligomers (Valensin et al., 2016; Shamoto-Nagai et al., 2018; Andersen et al., 2021). In consonance with these observations, recent comprehensive membrane lipid analyses with liquid chromatography-mass spectrometry have shown increases in unsaturated phospholipids, including PC, PE, and PS, in the amygdala and substantia nigra pars compacta of PD patient brains (Fu et al., 2022; Barbuti, 2024). Such changes were correlated with soluble α-syn accumulation and were associated with elevated lipid aldehydes, suggesting augmented lipid peroxidation. In addition, mitochondrial dysfunction is a well-established factor in PD pathogenesis, with studies showing impairments in mitochondrial respiratory chain complexes in PD patients (Erustes et al., 2022). However, research on mitochondrial phospholipids is limited. In PD, changes in cardiolipin levels, composition, and localization were indicated to contribute to mitochondrial function impairment and disease progression (Gilmozzi et al., 2020). Importantly, decreased levels of PE have been observed in the brains of early PD patients (Fanning et al., 2020), but it is unclear whether mitochondrial PE levels are affected.

Furthermore, studies have demonstrated that α-syn is associated with MAM (Guardia-Laguarta et al., 2014). Disruption of the α-syn-MAM association reduces ER-mitochondria contacts and increases mitochondrial fragmentation, highlighting the critical role of α-syn at the ER-mitochondria contact sites. It remains unclear whether α-syn is associated with MAM under PD-related pathophysiological conditions and whether the pathological form of α-syn species alters PE biosynthesis by impairing MAM function. In a study conducted in yeast with depletion of PE from the knockout of the gene encoding Psd1, heterologously expressed α-syn leads to abnormal accumulation of α-syn in the ER and an increase in ER stress. Administration of ethanolamine, convertible to PE through the ER CDP-ethanolamine pathway, largely mitigates the phenotypes of α-syn accumulation in the ER (Wang et al., 2014). Similar to the results in yeast, in a *C. elegans* PD model, α-syn-mediated dopamine neuron toxicity is exacerbated in the absence of PE production from mitochondria. Ethanolamine supplementation protects against dopamine neuron loss, which is attributed to PE production in the ER (Wang et al., 2014). Combined, these findings support the notion that PE homeostasis plays an important role in limiting cytosolic α-syn accumulation/aggregation and α-syn-induced neurotoxicity. However, how the reduction in PE supply from mitochondria disrupts α-syn homeostasis and thus augments its cellular toxicity remains largely unknown. Results from several earlier studies raise the possibility that PE may facilitate the association of α-syn with membranes through its role as a required substrate for glycosylphosphatidylinositol anchor biogenesis, thereby preventing cytosolic α-syn buildup and toxicity (Menon and Stevens, 1992; Birner et al., 2001; Chandra et al., 2003; Wang et al., 2014).

### Hereditary spastic paraplegia

HSP, also known as familial spastic paraparesis, refers to a group of inherited disorders that are characterized by upper motor neuron dysfunction that causes progressive muscle weakness and stiffness in the lower limbs, leading to difficulty walking. Strumpell and Lorrain first identified HSP in the late 19^th^ century and considered HSP as a small group of Mendelian disorders (Noreau et al., 2014; Statland et al., 2015). According to the accumulating understanding of its genetic architecture, this disorder has been recognized as one of the most genetically heterogeneous inherited disorders (Rickman et al., 2019). Aberrant PE biosynthesis has been linked to motor neuron diseases, especially HSP. Mutations in the EPT1 and PCYT2 enzymes involved in the CDP-ethanolamine pathway have been demonstrated to cause progressive spastic paraplegia, clinically overlapping autosomal recessive forms of complex HSP (Ahmed et al., 2017; Vaz et al., 2019). Additionally, mutations in genes that participate in the PC/PE metabolic cascade have also been associated with HSP. For example, DDHD1 and DDHD2 function as phospholipase A1 enzymes that break down phospholipids and are also involved in lipid transport and metabolism and vesicular trafficking (Tani et al., 2012). Mutations in *DDHD1* are associated with a range of motor neuron phenotypes that can be found in autosomal recessive HSP (SPG28) and juvenile amyotrophic lateral sclerosis (Bouslam et al., 2005; Miura et al., 2016; Wu and Fan, 2016; Dard et al., 2017), whereas mutations in *DDHD2* were shown to be related to an autosomal recessive early-onset spastic paraplegia (SPG54) (Schuurs-Hoeijmakers et al., 2012; Gonzalez et al., 2013). Importantly, *DDHD2* knockout mice also exhibited age-dependent apoptosis of motor neurons in the spinal cord (Maruyama et al., 2018). Therefore, mutations in a range of enzymes involved in PC/PE metabolism are linked to HSP, indicating that altered lipid metabolism and PC/PE imbalance are of great importance in maintaining motor neuron integrity and function.

In congruence with these observed genetic variants that are linked to disease phenotypes, decreased PE levels have been shown in lipidomics analysis of skin fibroblast cells from HSP patients and animal or cellular models of the disease (Horibata et al., 2018; Vaz et al., 2019; Kaiyrzhanov et al., 2021; Nunes et al., 2024). PS levels displayed decreased in patient fibroblasts with disease-causing PCYT2 variants (Vaz et al., 2019). As to alteration in PC levels, reductions in PC were observed in fibroblast cells from patients with HSP and yeast cells expressing SELENOI/EPT1 p.(Pro45Leu) variant (Vaz et al., 2019; Kaiyrzhanov et al., 2021), while increases in PC were found in mouse brains with SELENOI deficiency in the nervous system (Nunes et al., 2024). Given that mutations in PCYT2 have been linked to HSP (Ahmed et al., 2017; Kaiyrzhanov et al., 2021), researchers investigating PCYT2 deficiency proposed that supplementation with choline or serine may support alternative phospholipid biosynthetic pathways to help restore membrane phospholipid balance. This approach involves the production of PC through the CDP-choline pathway, the base exchange of PC to form PS by PSS1, and the subsequent production of PE through the PSD pathway (Vaz et al., 2019). Further assessment of the effects of choline and serine supplementation is essential to determine whether the increased availability of these nutrients would impact phospholipid levels.

## Therapeutic Potentials of Phosphatidylethanolamine and Phosphatidylserine Supplementations

Given that PE deficits are associated with neurological and other cellular dysfunctions and have been linked to neurodegenerative diseases, various approaches to increasing PE levels have been assessed. Because ethanolamine is a primary exogenous precursor for PE production through the CDP-ethanolamine pathway, ethanolamine supplementation may elevate PE levels. In fact, this view is supported by multiple lines of evidence from studies in yeast, *Drosophila melanogaster*, and mammalian cells. Indeed, the administration of ethanolamine was reported to enhance the endogenous PE pool and sufficiently extend the lifespan of yeast and *Drosophila melanogaster* (Rockenfeller et al., 2015). Moreover, in *psd1Δ* yeast, ethanolamine supplementation was found to increase PE levels via the CDP-ethanolamine pathway, which was associated with a reduction of ER stress without an impact on respiration (Wang et al., 2014). Such rescue effects are most likely attributed to restored PE species normally made in the ER but not those produced in mitochondria via the PSD pathway. In fibroblasts derived from spondyloepimetaphyseal dysplasia patients with homozygous missense variant c.797G>A/p.(Cys266Tyr) in *PISD*, administration of ethanolamine largely restored cell viability when these cells were under MG-132-induced stress (Girisha et al., 2019). However, as PE content was not assessed in these patient cells, it is unclear whether the protective effect of ethanolamine treatment results from enhanced PE supply. Importantly, the impact of ethanolamine supplementation on PE homeostasis has yet to be evaluated in animals and humans which should be a research objective for future endeavors.

Another approach to enhancing PE production could be stimulating PS decarboxylation via PSD in mitochondria. In yeast, overexpression of PSD1 was found to extend the lifespan and increase autophagy (Rockenfeller et al., 2015). As shown in a previous study in *Drosophila melanogaster* with a disruption of the CDP-ethanolamine pathway resulting from mutations in the *pect* gene, PSD overexpression can restore all species of cellular PE and fully rescue the abnormal phenotypes in these mutants. It was also demonstrated that this effect relies on lipid exchange between the ER and mitochondria (Zhao and Wang, 2020). While this approach needs to be tested in mammalian models, these findings show the induction of the PSD pathway in mitochondria as a promising method to correct deficiencies in the CDP-ethanolamine pathway in the ER. Moreover, suppressing the PEMT-mediated conversion of PE to PC could also increase tissue PE levels. Drugs that induce peroxisome proliferation or inhibit cellular methylation have been shown to produce such an effect in the liver without resulting in decreased PC levels due to compensatory increases in the activity of the CDP-choline pathway (Pritchard et al., 1982; Mizuguchi and Kawashima, 1996; Mizuguchi et al., 1999). Concerning AD treatment, peroxisome proliferators were shown to improve cognitive function (Biserni et al., 2008; Inestrosa et al., 2013). However, since these compounds do not appear to cross the blood-brain barrier, they may act through alternative mechanisms. Moreover, PEMT is much less abundant in the brain than in the liver (Blusztajn et al., 1979; Cui and Vance, 1996; Inestrosa et al., 2013). Thus, it remains unclear whether a reduction of PEMT would significantly impact PE homeostasis in the brain.

Additionally, many studies provided striking evidence showing that dietary supplementation of phospholipids can improve cognitive function. Notably, dietary supplementation with PE has been shown to extend lifespan in *C. elegans* by reducing insulin/IGF1-like signaling and enhancing DAF-16 activity (Park et al., 2021). However, there is a scarcity of data concerning the impact of the supplementation on cellular phospholipid content or composition (Delwaide et al., 1986; Engel et al., 1992; Cenacchi et al., 1993; Heiss et al., 1994; Hung et al., 2001; Moré et al., 2014). This could be attributable to limited approaches available for assessing these dynamic and highly regulated processes, including phospholipid metabolism, inter-organelle trafficking, and vesicular transport, particularly in live neurons. As a result, offering such critical information and the knowledge of the underlying mechanisms remain lacking. For instance, dietary PS increased blood PS levels, but whether the supplementation altered PE and other phospholipids in neural tissues is unknown (Delwaide et al., 1986; Engel et al., 1992; Cenacchi et al., 1993; Heiss et al., 1994; Moré et al., 2014). In senescence-accelerated mouse-prone 8 (SAMP8) mice, dietary supplementation with eicosapentaenoic acid-enriched PC and PE increased choline plasmalogen, lyso-PE, arachidonic acid-containing PE and PS in the cerebral cortex (Zhang et al., 2021). In rats, dietary PE was shown to be hydrolyzed to lyso-PE or ethanolamine before absorption into the circulation (Ikeda et al., 1987). Lyso-PE may be converted to PE through acylation or further degraded to form ethanolamine, which then enters the CDP-ethanolamine pathway for PE synthesis (Riekhof and Voelker, 2006). Therefore, further investigation is needed to directly assess whether and how dietary phospholipids are beneficial for restoring PE homeostasis in diseased brains.

Besides, recent studies have evaluated the therapeutic potential of supplementation of Omega-3 PUFAs found in PE and other phospholipids such as PC and PS in AD and PD (Hachem and Nacir, 2022). Omega-3 PUFAs have been indicated to have anti-inflammatory and neuroprotective potentials that may help protect against cognitive decline (Thomas et al., 2015; Wei et al., 2023). Two studies revealed that supplementation of omega-3 PUFA, particularly DHA and eicosapentaenoic acid, may be associated with a reduced risk of AD and cognitive decline (Wei et al., 2023; Castellanos-Perilla et al., 2024). Additionally, some research suggests that DHA may help reduce Aβ production and amyloid burden in AD brains (Cole et al., 2009; Heath and Wood, 2021). However, some clinical trials have yielded inconsistent results concerning the effectiveness of omega-3 PUFA supplementation in treating or preventing AD (Phillips et al., 2015; Wei et al., 2023). In PD, studies on the specific benefits of PE-PUFA supplementation remain very limited, and the available findings are inconclusive (Burckhardt et al., 2016; Yoo et al., 2021; Li and Song, 2022; Alves et al., 2024). Further investigations are required to fully understand the effects of omega-3 PUFA supplementation in AD and PD.

## Conclusions and Perspectives

Research on neurodegenerative diseases has long been centered on pinpointing the disease genes and their associations with disease-related pathologies. Emerging evidence on the connection of lipid dysregulation to neurodegenerative diseases urges us to fill the knowledge gap regarding its role in the pathogenesis of disease. The cellular regulation of lipid homeostasis is a complex process. PE plays an essential role in maintaining membrane integrity and dynamics and regulating proteins necessary for optimal mitochondrial respiratory function, among many other cellular functions. As most abundant membrane phospholipids in the brain, the importance of PE in neuronal function and survival has been established by a growing body of evidence, as reviewed in this article. Moreover, increasing findings concerning mutations in genes encoding enzymes involved in PE metabolism in association with neurological disorders also indicate the significance of PE imbalance in the pathogenesis of these conditions (St Germain et al., 2022). Indeed, PE deficiency is a common feature in major neurodegenerative diseases. However, a basic understanding of the mechanism underlying the maintenance of phospholipid homeostasis in neurons remains extremely limited. Many questions remain: How do the PSD and CDP-ethanolamine pathways interplay in neurons? How do mitochondria contribute to PE supply in neurons? As a primary constituent of SVs, is PE involved in regulating neurotransmission by modulating SV biogenesis? What is the mechanism underlying PE metabolic defects in diseased neurons? What is the role of mitochondrial dysfunction in PE deficiency in the disease? How does PE imbalance influence disease-related pathologies? Does restoration of PE homeostasis yield beneficial effects in neurodegenerative diseases? What is the therapeutic potential for PS and PE supplementations in these diseases?

Several salient themes in the present research progress are worth highlighting, which may shape directions for future efforts on the role of PE biosynthetic dysfunction in neurodegenerative diseases. First, among many other aspects where neurons differ from non-neuronal cells, neurons are featured with characteristic morphology and long-distance axonal transport involved in membrane trafficking, lipid-protein interactions, and organelle function. As much of the current understanding of PE has come from research in non-neuronal cells and other organisms (e.g., yeast), future studies need to gain more mechanistic insights into PE metabolism and production in neurons and their impacts on neuronal health. Second, the interplay of two primary PE biosynthetic pathways via mitochondria and the ER and PE exchange between these two organelles are crucial for coordinating PE production and supply to membranes as well as serving as compensatory mechanisms upon PE deficits in the disease. However, a detailed understanding of such mechanisms remains lacking in neurons. Systematic efforts are critically needed to assess the relative contribution of each pathway and the extent of their mutual compensation in supporting neuronal function under physiological and pathophysiological conditions. Data from these investigations are expected to promote the development of new strategies to modify PE production and trafficking and thus improve treatment outcomes. Third, as PE metabolism/biosynthesis is an energy-requiring process, functionally distressed mitochondria may further exacerbate disease-linked PE synthesis deficits. This notion has garnered some support from recent studies in tauopathy neurons (Jia et al., 2025), suggesting that stimulating mitochondrial bioenergetics and increasing PE levels in a synergistic manner could be more effective than a single PE-targeted intervention. Therefore, more work is warranted to advance our understanding of the interplay between mitochondrial dysfunction and PE imbalance and its implications in AD and other neurodegenerative diseases. Fourth, multiple lines of evidence have linked PE biosynthesis failure to disruptions in mitochondrial respiration. Given that mitochondrial dysfunction is a central problem in neurodegenerative diseases (Cai and Jeong, 2020), PE synthesis defects may be involved in forming a downward spiral in mitochondrial deterioration. Thus, many of the detailed mechanisms underlying such defects need to be deciphered, including the role of PE in the assembly and activity of supercomplexes in the electron transport chain embedded in the IMM (Decker and Funai, 2024). Fifth, compelling evidence from multiple studies in *C. elegans* and mice has demonstrated that major PE species are significantly decreased in aged organisms, highlighting PE reduction as a pronounced feature of aging (Dai et al., 2021). Such changes may augment the vulnerability of neurons under pathophysiological conditions and consequently exacerbate neuropathology and functional defects in age-related neurological diseases such as AD and PD (Calzada et al., 2016). Thus, more investigations on PE metabolism in aging neurons are of great importance to advance our understanding of the role of PE deficiency associated with these diseases. Furthermore, assessing the changes in the PSD or CDP-ethanolamine pathways during normal aging and at different stages of the disease may help discover new mechanisms beneficial for future therapeutic development. Lastly, most studies of PE metabolism in diseases have been centered on determining the changes in the levels of PE and other phospholipids. Future investigations need to focus more on dissecting the mechanisms underlying such changes. Also, the field will drastically benefit from the research on assessing the effectiveness and feasibility of the treatment options for normalizing PE deficits and evaluating their effects against disease-related pathologies. Advances in these areas in the coming years are expected to facilitate the identification of novel mechanisms in neurodegenerative diseases and the development of new therapeutic strategies to treat these diseases.

## Data Availability

*Not applicable*.
